# Fluorescence Visual Detection of Herbal Product Substitutions at Terminal Herbal Markets by CCP-based FRET technique

**DOI:** 10.1038/srep35540

**Published:** 2016-10-21

**Authors:** Chao Jiang, Yuan Yuan, Guang Yang, Yan Jin, Libing Liu, Yuyang Zhao, Luqi Huang

**Affiliations:** 1 State Key Laboratory Breeding Base of Dao-di Herbs, National Resource Center for Chinese Materia Medica, China Academy of Chinese Medical Sciences, Beijing, 100700, P. R. China; 2 Beijing Area Major Laboratory of Protection and Utilization of Traditional Chinese Medicine, College of resources, Beijing Normal University, Beijing, 100875, P. R. China; 3 Beijing National Laboratory for Molecular Sciences, Key Laboratory of Organic Solids, Institute of Chemistry, Chinese Academy of Sciences, Beijing, 100190, P. R. China

## Abstract

Inaccurate labeling of materials used in herbal products may compromise the therapeutic efficacy and may pose a threat to medicinal safety. In this paper, a rapid (within 3 h), sensitive and visual colorimetric method for identifying substitutions in terminal market products was developed using cationic conjugated polymer-based fluorescence resonance energy transfer (CCP-based FRET). Chinese medicinal materials with similar morphology and chemical composition were clearly distinguished by the single-nucleotide polymorphism (SNP) genotyping method. Assays using CCP-based FRET technology showed a high frequency of adulterants in Lu-Rong (52.83%) and Chuan-Bei-Mu (67.8%) decoction pieces, and patented Chinese drugs (71.4%, 5/7) containing Chuan-Bei-Mu ingredients were detected in the terminal herbal market. In comparison with DNA sequencing, this protocol simplifies procedures by eliminating the cumbersome workups and sophisticated instruments, and only a trace amount of DNA is required. The CCP-based method is particularly attractive because it can detect adulterants in admixture samples with high sensitivity. Therefore, the CCP-based detection system shows great potential for routine terminal market checks and drug safety controls.

Herbal medicines have a long and well-documented history, having been used for thousands of years in traditional Oriental, Ayurvedic, and Latin American medicine to prevent chronic diseases and improve health. The global resurgence in traditional treatments is expanding the herbal commodity market^1^. However, for historical and geographical reasons, closely related species share similar morphological appearances, textures and microscopic characteristics, and these species form contaminants, adulterants, and counterfeits in herbal commodity markets. As highlighted in recent reports, a survey of the species composition of commercial herbal products revealed considerable product adulteration and ingredient substitution in North America, Hong Kong and Morocco terminal herbal markets^2^,^3^,^4^. For certain species, an even higher frequency of substitution appears in turmeric powder (20%)^5^, *Ginkgo biloba* leaf (24.3%)^6^, black cohosh dietary supplements (25%)^7^, commercial teas (33%)^8^, and *Rhodiola* decoctions (60%)^9^. Similar to the situation for herbal dietary supplements, substitutions and counterfeits have also been documented in patented Chinese drugs^10^,^11^,^12^. Moreover, due to their heterogeneous origins and differences in preparation methods, the substitutes for patented Chinese drugs may be present at higher levels than indicated by current estimates.

Inaccurate labeling of herbal products may compromise therapeutic efficacy and may pose a threat to medicinal safety. For instance, aristolochic acids, a major toxic ingredient in some herbal materials, cause serious toxicity in traditional medicine clinics^13^,^14^,^15^. Investigations revealed that the most lethal cases were caused by the mislabeling of non-harmful herbal materials that were replaced with morphologically similar poisonous species in the herbal market^4^,^16^,^17^. This substitution causes increasing consumer concern about the authenticity of the products they purchase. However, the accurate authentication of herbal medicines is difficult for consumers due to the lack of taxonomical knowledge. Moreover, to enable quick use, herbal products at terminal herbal markets typically are extensively processed and prepared as decoction pieces, herbal dietary supplements or patented Chinese drugs; because of the resulting lack of adequate botanical identification characteristics, contamination or adulteration can easily occur.

The present prevailing authentication platforms are based on phytochemical identification. Most of these chemical identification methods (e.g., high performance liquid chromatography, gas chromatography, and mass spectrometry) are based on detecting the characteristic chemical constituents, thus providing “dominant marker” methods that can only identify the main components and are unable to recognize contaminations and admixtures^18^. Furthermore, substitutes are generated using species that are closely related to the authentic product and are thus difficult to distinguish because of the shared chemical structures. The efficacy of chemical methods is further limited when applied to admixtures containing closely related species. To improve the accuracy of identification, DNA markers, particularly single-nucleotide polymorphisms (SNPs), have been used to differentiate plants at the species level^19^. More advanced than other molecular markers, SNPs are biallelic and can be used to distinguish heterozygous genotypes or admixtures through comparatively simpler methods. Because only trace amounts of materials are included in processed products, a simple and sensitive method for identifying these materials is required to approximate the ease of visual inspection methods.

In recent years, with the advantage of the fluorescence signal amplification of cationic conjugated polymers (CCPs) to increase the sensitivity for DNA detection, electrostatic complexes of CCPs with DNA were designed as optical biosensors. These complexes offer a convenient and cost-effective fluorescence assay for SNP genotyping^19^,^20^,^21^,^22^,^23^. Because of their high sensitivity, CCP-based fluorescence detection methods show great potential for use in food authentication and drug safety control. Herein, we demonstrate a homogeneous, cost-effective, and high-throughput method that combines CCP and fluorescence resonance energy transfer (FRET) to discriminate SNP genotypes and screen for species origin of terminal herbal market products. The application of this method to origin screening was validated by detecting two famous traditional Chinese medicinal products, bulbus fritillariae cirrhosae (Chuan-Bei-Mu) and deer velvet (Lu-Rong) from terminal herbal markets. This detection system revealed a high frequency of adulteration of Chuan-Bei-Mu or Lu-Rong with closely related species in decoctions and patented Chinese drugs. Furthermore, the improved detection of mixture samples lowered the false positive rate compared with that of other dominant marker detection approaches^24^. Therefore, the CCP-based detection system improves routine terminal market checks and drug safety control.

## Results

### Principles of CCP-based fluorescence authentication

**Figure 1:**
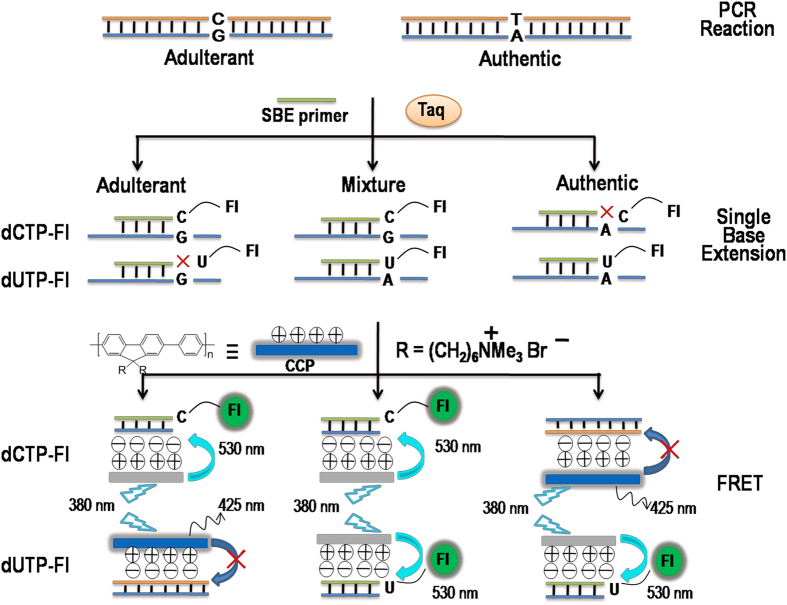
Schematic of the principle of CCP-based fluorescence genotyping of SNPs for the authentication of botanical origin. The target DNA fragment contains a single-nucleotide polymorphism (SNP) site (G > A). Fluorescein-labeled dCTP (dCTP-Fl) or fluorescein-labeled dUTP (dUTP-Fl) was incorporated into the 3′-terminal of the specific extension primer by a single-base extension reaction that is only present for the G or A allele-containing PCR fragment, respectively. Thus, by triggering a fluorescence resonance energy transfer (FRET) signal from poly{(1,4-phenylene)-2,7-9,9-bis(6′-*N*,*N*,*N*-trimethyl ammonium)-hexyl fluorene. dibromide} (PFP) to fluorescein, the SNP genotypes are discriminated.

Figure 1 shows the principle of the CCP-based fluorescence genotyping of SNPs for the authentication of deer velvet and its adulterants. A sequence that contains a polymorphic site is used as a DNA target for which the nucleotide A in the wild type (Authentic deer velvet) is replaced by G in the mutant target (Adulterant). Thus, three SNP genotypes are possible: homozygous A (Authentic), homozygous G (Adulterant), and heterozygous G/A (Mixture). The DNA fragments in cytochrome c oxidase subunit I (COI) were amplified by PCR, and then these PCR products were used as a template for single-base extension (SBE) reactions to incorporate fluorescein-labeled dNTP (dNTP-Fl) at the 3′ end of the specific extension primer. A water-soluble CCP was added to the reaction mixture; thus, FRET from CCP to fluorescein would only occur if the dNTP-Fl was an exact match to the SNP sites. In the FRET experiments, a water-soluble CCP, poly{(1,4-phenylene)-2,7-9,9-bis(6′-*N*,*N*,*N*-trimethyl ammonium)-hexyl fluorene. dibromide} (PFP), was used as the donor, and fluorescein-labeled dCTP (dCTP-Fl) and fluorescein-labeled dUTP (dUTP-Fl) were chosen as the acceptors. PFP acts as the donor for fluorescein, and fluorescein acts as the acceptor for PFP to satisfy the overlap integral requirement for FRET^25^. The primer was complementary to the wild-type target, and the mutant target was immediately upstream of the polymorphic site. Taq DNA polymerase and either dCTP-Fl or dUTP-Fl were used for primer extension reactions. For the reaction using dCTP-Fl, dCTP-Fl was incorporated into the 3′-terminal of the specific extension primer only in the presence of allele G. Upon the addition of PFP, strong electrostatic interactions between the negatively charged DNA and the cationic PFP decreases their separation, and efficient FRET occurs from PFP to Fl. Similarly, for the reaction using dUTP-Fl, dUTP-Fl was incorporated into the 3′-terminal of the specific extension primer only in the presence of allele A, and efficient FRET from PFP to Fl occurred. In this case, for the excitation of PFP at 380 nm, the SBE product of authentic samples (homozygous A allele) can only generate FRET in the presence of dUTP-Fl. The SBE product of adulterated samples (homozygous G allele) only produces FRET in presence of dCTP-Fl, and the mixture (heterozygous G and A) could produce FRET in the presence of either dUTP-Fl or dCTP-Fl. The weak electrostatic interactions between dCTP-Fl, dUTP-Fl and CCP can not keep them sufficiently close in the no-template control (NTC), which results in inefficient FRET from CCP to Fl. Through the shift in emission color or the change in the emission intensity of PFP and fluorescein, it is possible to detect four possible statuses (two homozygous, one heterozygous and NTC) in the extension reaction.

### CCP-based assay method for the authentication of herbal medicinal

**Figure 2:**
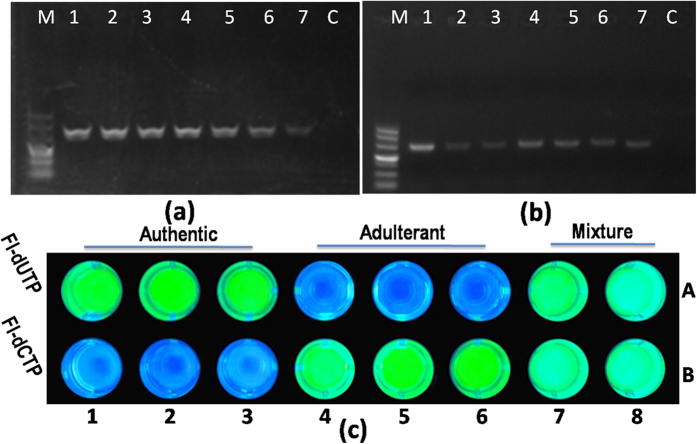
(**a**) PCR amplification of deer velvet using the specific primer pair LR-F/LR-R. The lanes 1–7 were Sika deer (*Cervus nippon*), red deer (*C. elaphus*), sambar (*C. unicolor*), reindeer (*Rangifer tarandus*), white-lipped deer (*C. albirostris*), Pere David’s deer (*Elaphurus davidianus*) and fallow deer (*Dama dama*), respectively. (**b**) PCR amplification of fritillaria using the specific primer pair CB-F/CB-R. Lanes 1–7 were *Fritillaria cirrhosa*, *F. unibracteata*, *F. delavayi*, *F. przewalskii*, *F. taipaiensis*, *F. wabuensis* and *F. walujewii*, respectively. C was a no-template control (NTC) and was used as the blank. Electrophoresis was performed on a 2% agarose gel, which was stained with GeneGreen. The band sizes of the DL500 DNA marker are indicated on the left. (**c**) A photograph of the fluorescence pattern of deer velvet and its adulterants on a microtiter plate. SBE products mixed with CCP (15 μmol·L^−1^ in RUs) in PCR tubes under 365 nm UV light irradiation. Species: 1–2: *C. nippon*; 3: *C. elaphus*; 4: *C. unicolor*; 5: *R. tarandus*; 6: *E. davidianus*; 7–8: mixture of *C. elaphus* and *R. tarandus*.

The origin screening of deer velvet and fritillaria was performed using incorporated dye-dNTP for dUTP-Fl for authentic fritillariae cirrhosae and dGTP-Fl for the adulterant. In this case, the deer velvet DNA fragments with a length of 424 bp (Fig. 2a) and fritillaria DNA fragments with a length of 275 bp (Fig. 2b) were amplified by PCR, and then, the remaining primers and unincorporated dNTPs were removed using 5 μL of Exo-SAP regent and 0.5 U of pyrophosphatase (YIPP). An SBE reaction was performed in parallel using dUTP-Fl or dCTP-Fl, and after the SBE reaction, CCP was added as the FRET donor for 365 nm ultraviolet light irradiation. As indicated in Fig. 2c, green fluorescence was emitted for the corresponding complementary genotype. These results indicate that it is possible to visibly genotype SNPs with the naked eye through the CCP-based fluorescence assay method.

**Figure 3:**
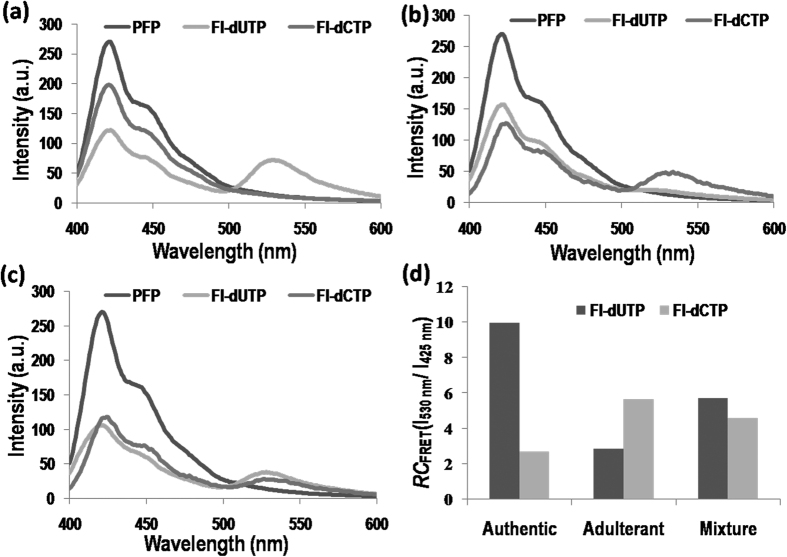
Fluorescence assay results for authentication of Lu-Rong and its adulterants using the CCP-based SNP genotyping method, for which a single base extension reaction was conducted using dCTP-Fl or dUTP-Fl in parallel. Emission spectra: (**a**) authentic Lu-Rong exhibits a homozygous A allele; (**b**) the adulterant exhibits a homozygous G allele; and (**c**) the mixture exhibits a heterozygous G/A allele. (**d**) Relative fluorescence resonance energy transfer (FRET) ratios of PFP to fluoresceinRC_FRET_(I_530 _nm/I_425 _nm). For homozygous A, homozygous G and heterozygous G/A.

A fluorescence emission spectrum was also measured under 380 nm ultraviolet light in transmission mode, as shown in Fig. 3. The maximum emission of PFP itself in buffer solution appeared at approximately 425 nm, and no fluorescein emission was observed at 530 nm. For the sample containing complementary dNTP-Fl for the SBE reaction, efficient FRET between PFP and fluorescein led to significant quenching of the PFP emission at 425 nm and to the appearance of fluorescein emission at 530 nm for both the authentic and adulterant samples (Fig. 3a,b). For the mixture samples containing both the heterozygous G and A genotypes, fluorescein emission at 530 nm was found in both the dUTP-Fl and dCTP-Fl samples (Fig. 3c). The FRET ratios (ratio of the emission at 530 nm to that at 425 nm) were used to discriminate authentic samples from its adulterants. As defined in Equation (1), FRET and FRET_0_ are the relative changes in the FRET ratio for the initial PFP-added and target-added reactions, respectively. Through measurements of the relative ratio of the emission intensities of the conjugated polymers and fluorescein, the SNP genotypes can be clearly discriminated (Fig. 3d).


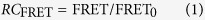


**Figure 4:**
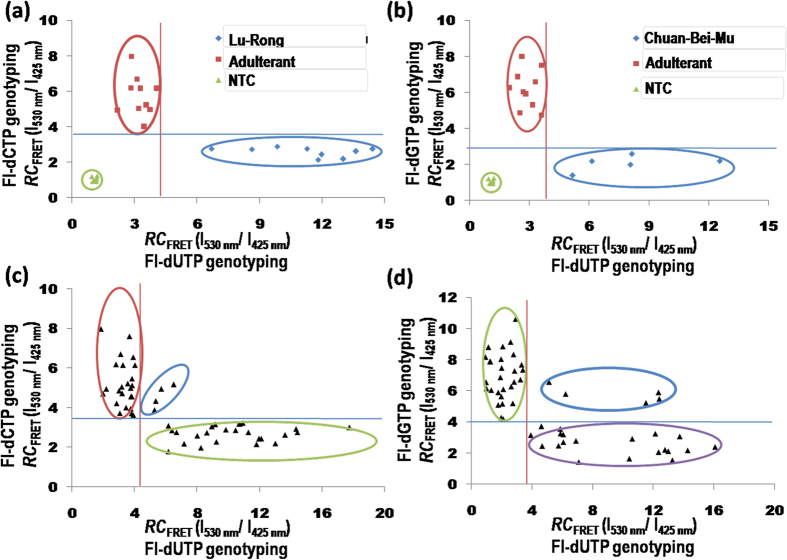
Genotyping results for the authentication of Lu-Rong and Chuan-Bei-Mu using the CCP-based SNP genotyping method. A no-template control (NTC) is used as a blank. (**a**) Relative fluorescence resonance energy transfer (FRET) ratio of PFP to fluoresceinRC_FRET_ (I_530 _nm/I_425 _nm). In Lu-Rong and its adulterants using Fl-dCTP (x axis) and Fl-dUTP (y axis) in an SBE reaction. (**b**) RC_FRET_ (I_530 _nm/I_425 _nm) in Chuan-Bei-Mu and its adulterants using the Fl-dUTP (x axis) and Fl-dGTP (y axis) in an SBE reaction. CCP-based SNP genotyping authenticated the results of 53 specimens of Lu-Rong (**c**) and 59 specimens of Chuan-Bei-Mu (**d**) collected from terminal herbal markets. Blue, red and green circles are the authentic, adulterant and the mixture samples, respectively, that were identified using the present method.

The scatter diagram for genotyping to determine the botanical origin of Lu-Rong and Chuan-Bei-Mu was plotted by measuring the relative ratio of the emission intensitiesRC_FRET (dNTP)_. Using the corresponding complementary fluorescein-labeled dNTP in an SBE reaction. The threshold values for authenticity were determined to be RC_FRET (dUTP)_ > 3.5 and RC_FRET (dCTP)_ < 3.7 for Lu-Rong and RC_FRET (dUTP)_ > 3.3 and RC_FRET(dGTP)_ < 3.8 for Chuan-Bei-Mu (Fig. 4a,b). Through these threshold values, all authentic samples were separated from their adulterants.

### Sensitivity and Specificity

The sensitivity, specificity and diagnostic accuracy of the CCP-based FRET method with 95% confidence intervals (CI) were calculated by using the PCR-RFLP (a standard method recorded in China pharmacopoeia to identify Chuan-Bei-Mu) as the reference standard. 38 of 39 specimens (97.4%; 95% CI: 96.8–99.6%) met the result of PCR-RFLP, while 1 specimens (2.5%) failed to meet threshold values. In terms of specificity, all negative specimens (42 indivaduals) met the result of PCR-RFLP (95% CI: 91.7–100%) and the diagnostic accuracy was 98.8% (95% CI: 93.3–99.8%). The identify results of CCP-based FRET and PCR-RFLP were also demonstrated in Fig. S3 (Supplementary Information).

Due to the high sensitivity and specificity, the method of CCP-based FRET was also employed to reveal adulterants in herbal medicines. Fifty-three decoction pieces specimens labeled as Lu-Rong and fifty-nine decoction pieces specimens labeled as Chuan-Bei-Mu were collected from terminal herbal markets and then genotyped through the present method. As demonstrated in Fig. 4c,d, high proportions of Lu-Rong (52.83%) and Chuan-Bei-Mu (67.8%) decoction pieces were determined to be potentially mislabeled. These results were confirmed by morphological authentication or DNA sequencing (Table 1). Comparison of the data obtained from these methods revealed that the positive rate of CCP-based FRET is comparable to those of DNA sequencing (52.83% vs. 48.0%) and morphological authentication (67.8% vs. 67.8%). Patented Chinese drug sample identification. Patented Chinese drugs are an important part of terminal herbal markets. Inadequate information about the morphological texture and ambiguous chemical signatures make it difficult to determine the crude drug origin using traditional authentication methods. To circumvent these deficiencies, CCP-based FRET was used to reveal mislabeling in patented drugs. Sixteen patented Chinese drugs containing the crude ingredients of Chuan-Bei-Mu or it sibling species Zhe-Bei-Mu were screened for their origins using this detection system. As depicted in Table 2, only two patented drugs labeled as Chuan-Bei-Mu in a dispensatory exceeded the relative FRET ratio threshold and were deemed authentic, whereas three drugs were deemed substitutes that contained sibling species, and two were considered a mixture of Chuan-Bei-Mu and other sibling species, as determined by the CCP-based FRET approach. Furthermore, the value of the relative FRET ratio for all the drugs labeled as Zhe-Bei-Mu in a dispensatory fell into the category of non-Chuan-Bei-Mu; this category exhibited the homozygous C genotype for this allele, indicating a Zhe-Bei-Mu botanical origin. Four patented drugs in the analyzed samples were negative and showed the absence of a PCR-amplified product band on the GeneGreen-stained agarose gel; this result may be caused by low-quality DNA or a lack of fritillaria ingredients. Therefore, after the elimination of the uncertain samples, five drugs labeled as Chuan-Bei-Mu in a dispensatory were determined to be potentially mislabeled, showing a high rate of substitution in patented Chinese drugs.

## Discussion

In light of the critical requirement for convenient identification of ingredients and contaminants, a rapid fluorescence method for identifying medicinal species is highly desirable. Combining FRET with CCPs, we developed a rapid method for SNP genotyping of extracted DNA with SBE to detect the species origins in herbal medicines that are currently available on the terminal market. A water-soluble CCP, PFP, was used as the donor in the FRET experiments. Fluorescein-labeled dNTP was chosen as the acceptor. Through an emission change in the fluorescein intensity or the color of the solution, it was possible to detect four possible outcomes (two homozygous genotypes, a heterozygous genotype and a blank status) in the extension reaction. It is important to simultaneously differentiate homozygous and heterozygous genotypes during counterfeit detection because the numerous authentic samples in an admixture may cause false positive results. In this study, we determined the detection limit of the proposed CCP-based FRET method for Chuan-Bei-Mu and its adulterants. By using various percentage mixtures of authentic (*Fritillaria cirrhosa*) and adulterant (*F. ussuriensis*) genomic DNA, followed by PCR amplification and then an SBE reaction, mutant DNA at percentages as low as 5% was detected (Figs S1 and 2, Supplementary Information). Moreover, in contrast to the situation for conventional morphological identification or DNA sequencing, regression curves of the FRET ratio and the two serial percentage DNA experiments used in this method could be constructed to provide quantitative assessments of the adulteration percentage. The quantitative assay with single-base resolution is useful for differentiating closely related species in herbal medicines, which share similar morphological characteristics and are predominant adulterants in herbal commodity markets.

**Figure 5:**
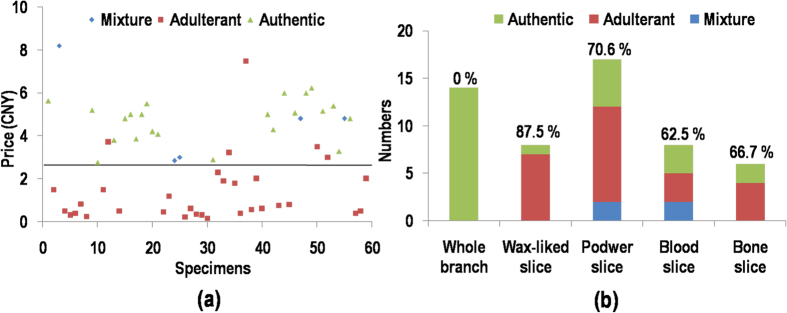
(**a**) Relation between retail prices and the CCP-based FRET authentication results in the Chuan-Bei-Mu decoction pieces. (**b**) Commercial specification and the CCP-based FRET authentication results of the Lu-Rong decoction pieces.

In the present study, the CCP-based FRET assay showed that a large fraction (approximately 50–70%) of the herbal products incorporated adulterant-specific Fl-dNTP during the SBE reaction and emitted fluorescence at 530 nm; this result indicated that these herbal products contained mislabeled ingredients. The finding of mislabeling in multiple products suggests the possibility of counterfeits or substitutions to improve the appearance or for economic reasons. Interestingly, the Chuan-Bei-Mu decoctions were associated with economic incentives based on differences in retail prices, whereas the products priced lower than 2.5 CNY (Chinese Yuan) were identified as adulterants or admixtures (Fig. 5a). In instances that lacked an economic incentive, accidental mishandling by the manufacturer or the supplier may have resulted in the listed product being replaced by higher-valued species because the substitution would have resulted in profit loss. For the Lu-Rong decoction pieces, the adulterant ratio was slightly lower than for Chuan-Bei-Mu (52.83% vs. 67.8%), and the adulteration rate was not obviously related to its retail price. However, when the Lu-Rong decoction pieces were classified into five specifications, whole branch (WB), wax-liked slice (WS), powder slice (PS), blood slice (BlS) and bone slice (BoS), a remarkable adulterant frequency difference emerged between the specifications. The WB velvet sample was completely authentic, and the substitutions mainly contained WS and PS (Fig. 5b), possibly because the morphological features of WB deer velvet were no longer discernable after the complicated processing procedure. Other studies also demonstrated a high fraud ratio in the processing of deer products^26^,^27^.

Another advantage of our CCP-based FRET method is the ability to quickly differentiate herbal medicines from closely related adulterants in patented Chinese herbal drugs, thereby guaranteeing the clinical drug safety and protecting the vital interests of patients. In contrast to the usual strategies based on PCR that detect the authentic ingredient-derived DNA contained in the products, our CCP-based FRET method amplifies specific consensus DNA fragments of an authentic product and its adulterants with an SNP allele. PCR amplification bias was avoided, and non-target DNA ingredients from other constituents were eliminated. Due to the high specificity in the SBE reaction, this method provides good selectivity for the identification of fritillaria in patented Chinese drugs and is sufficiently robust for high-throughput screening. Therefore, this CCP-based FRET method shows great potential for routine terminal market checks of crude material, decoction pieces, dietary supplements and patented drugs. This technology represents a direct application that is appropriate for supervisory institutions and for protecting the legitimate rights and interests of the consumers.

## Conclusion

We developed a visual colorimetric method for the rapid detection of SNP mutations by combining FRET with water-soluble CCPs. SNP genotyping can be achieved by directly observing the emission color change of the assay solution in a 96-well PCR plate under UV light irradiation. Using assays from this CCP-based FRET technology, a high frequency of adulterants in Lu-Rong (52.83%), Chuan-Bei-Mu (67.8%) decoction pieces and patented Chinese drugs (71.4%, 5/7) containing Chuan-Bei-Mu ingredients was revealed in terminal herbal market products. In comparison with DNA sequencing, this protocol simplifies procedures by eliminating cumbersome workups and sophisticated instruments, and only a trace amount of DNA is required. The CCP-based method is particularly attractive because it can detect adulterants in admixtures with high sensitivity. The method can also be used to authenticate other substitutions of closely related species in routine terminal market checks and shows great potential for applications in drug safety control.

## Materials and Methods

### Herbal material

Fritillaria and deer antler reference specimens or validation samples were obtained from various regions of the Anhui and Beijing provinces, China (Table S1, Supplementary Information). Dried bulbus fritillariae cirrhosae (Chuan-Bei-Mu) and deer velvet (Lu-Rong) samples were collected from different pharmacies, drug markets, companies or hospitals from China (Tables S2 and S3, Supplementary Information). Reference and validation samples were morphologically identified by the authors. Sixteen patented Chinese drugs were purchased in retail stores and herbal markets in Beijing, China. (Table S4, Supplementary Information). The herbal supplements consisted of dry, cut, and sifted plant materials (gelatin capsules, honey pills or compression tablets).

### Genomic DNA extraction

Materials were frozen in liquid nitrogen and ground to a fine powder with a MM 400 Mixer Mill (Retsch Technology GmbH, Haan, Germany). At least 3 g of material was powdered, and approximately 25 mg of powder was randomly selected for DNA extraction. The fritillaria DNA was extracted using the cetyltrimethylammonium bromide (CTAB) method. The deer antler DNA was extracted using a DNeasy Blood & Tissue Kit (QIAGEN, Valencia, CA) according to the manufacturer’s protocol and stored at −20 °C. The concentration of the isolated DNA and the ratio of the absorbances at 260 nm to 280 nm (OD_260_/OD_280_ ratio) were measured with a NanoDrop ND-1000 spectrophotometer (Gene, Hong Kong, China). Patented Chinese drugs contained fritillaria ingredients were also tested in this study. Approximately 100 mg of patented drug powders were randomly selected for DNA extraction. The DNA was extracted using the modified CTAB method, and therefore, the DNA was purified with the GeneJET Gel Extraction Kit (Thermo Scientific) following the standard protocol.

### PCR amplification

PCR was performed to amplify gene fragments that included the mutation sites of interest. PCR amplification was performed in a total volume of 25 μL containing 2.5 μL of 10× Fast Buffer I (Takara, China), 1 μL of dNTP Mixture (2.5 mol·L^−1^ each, Takara), 0.2 μL of SpeedStar HS *Taq* DNA polymerase (5 U·μL^−1^, Takara), 0.2 μL each of the forward and reverse primers (10 μmol·L^−1^, Sangon), and approximately 10 ng of genomic DNA. Thermal cycling was performed in a Veriti thermocycler with rapid PCR: an initial denaturation at 94 °C for 2 min, followed by 30 cycles of 5 s at 94 °C and 10 s at 60 °C, and a final extension of 2 min at 72 °C. After the cycle reaction, the reaction products were held at 4 °C. The PCR products were checked for quality and for yield by running 5 μL in 2% agarose gels in Tris-Acetate-EDTA solution. Next, 10 μL of the PCR products was used for treatment with 5 μL of ExoSAP-IT^®^ Clean-Up reagent (Affymetrix, Cleveland, USA) and 0.05 units of pyrophosphatase at 37 °C for 1 h to remove the excess primers, dNTPs and pyrophosphate generated during PCR and heated at 80 °C for 10 min to inactivity. Negative control reactions (using ddH_2_O instead of genomic DNA) were performed simultaneously to ensure that the PCR products were not the result of contamination of the reagents with DNA.

### Single-base primer extension

Single-base primer extension mixtures (25 μL) were prepared and included 2.5 μL of 10× Fast Buffer I, 10 pM of the corresponding fluorescein-12-dNTP, 2.5 μL of specific SBE primer (10 μmol·L^−1^), 0.3 μL of SpeedStar HS *Taq* DNA polymerase (5 U μL^−1^), and 14.2 μL of high-purity water. The mixtures were pipetted into adjacent columns of a 0.2-mL, 96-well PCR plate on ice. Five microliters of digested samples was added individually to the appropriate wells of the PCR plate. The reaction was performed in a Veriti^®^ Thermocycler as follows: initial denaturation at 94 °C for 1 min; 25 cycles of denaturation at 94 °C for 5 s, annealing and extension at its annealing temperature (Table S6, Supplementary Information) for 5 s, and holding at 4 °C. The SAP solution included 3 μL of 10× SAP buffer, and 2 μL of shrimp alkaline phosphatase (0.5 U·μL^−1^, New England Bio., Beijing, China) was prepared and added to each well of the 96-well PCR plate containing the single-base primer-extension products. The plate was transferred to a thermocycler and incubated at 37 °C for 20 min, heated at 80 °C for 10 min, and held at 4 °C.

### Fluorescence detection

For fluorescence detection using the fluorescence resonance energy transfer (FRET) ratio, 80 μL of 25 mM HEPES buffer and 20 μL of 15 μmol·L^−1^ PFP were added to the 96-well microtiter plates (Thermo Scientific). The mixture was vortexed vigorously for 5 s, and the emission spectra or the intensity of the solution was measured by a Varioskan Flash Spectral Scanning Reader (Thermo Scientific) equipped with an excitation filter of 380/5 nm. Upon the addition of 20 μL of SBE products, the spectrum was measured again. The emission filters were 440/5 nm for PFP and 530/5 nm for fluorescein. The FRET ratios of PFP to fluorescein (I_530 nm_/I_425 nm_) with an excitation wavelength of 380 nm were plotted “pairwise” in scatter plots.

For visible detection of FRET, 20 μL of PFP was added to PCR tubes or the 96-well PCR plate containing the single-base primer-extension products and mixed thoroughly by pipetting. Photographs were taken in a WD-9403F UV Viewing Cabinet under 365 nm UV light irradiation, and a digital camera (Pentax k7) was used to record the images. The contrast of the images was adjusted so that the inherent fluorescence of the PCR plate was not visible. For the imaging of the 96-well PCR plate, it was better to position the 96-well PCR plate upside down to obtain uniform UV light irradiation on the SBE product/CCP mixture.

### PCR-RFLP analysis

Twenty Chuan-Bei-Mu decoction piece samples (CB0001~CB0020) and 61 unidentified Chuan-Bei-Mu samples from An’hui Institute for Food and Drug Control were conducted by both PCR-RFLP analysis and the new established CCP-FRET assay to test its sensitivity and specificity. PCR was performed in a total volume of 30 μL with forward primer 5′-CGTAACAAGGTTTCCGTAGGTGAA-3′ and reverse primer 5′-GCTACGTTCTTCATCGAT-3′. PCR amplification system and thermal cycling was accord to the China pharmacopoeia Part I. PCR-RFLP mixtures (20 μL) were then prepared in PCR plate and included 2 μL of 10× digest Buffer I, 0.5 μL of Sma I endonuclease (New England Bio., Beijing, China), 15 μL of PCR products, and 2.5 μL of high-purity water. The plate was transferred to a thermocycler and incubated at 25 °C for 120 min, heated at 80 °C for 10 min, and held at 4 °C. PCR-RFLP products were further analysed by 1.5% agarose gel (Invitrogen, California, USA) electrophoresis with GelRed dye (TIANGEN, Beijing, China) in 1× TAE buffer.

## Additional Information

**How to cite this article**: Jiang, C. *et al.* Fluorescence Visual Detection of Herbal Product Substitutions at Terminal Herbal Markets by CCP-based FRET technique. *Sci. Rep.* **6**, 35540; doi: 10.1038/srep35540 (2016).

## Supplementary Material

Supplementary Information

## Figures and Tables

**Table 1:** Summary of CCP-based FRET, morphological authentication, and DNA sequencing results for the Lu-Rong and Chuan-Bei-Mu decoction pieces.

Methods	Lu-Rong (N = 53)			Chuan-Bei-Mu (N = 59)		
	Authentic	Adulterant	Mixture	Authentic	Adulterant	Mixture
CCP-based FRET	25	24	4	20	34	5
morphological/sequencing*	26	24	0	20	36	3

A threshold value was established as the mean ± 3 s.t.d. of 20 samples, and a sample was deemed positive if the FRET ratio exceeded this value. The threshold values for authenticity are RC_FRET (dUTP)_ > 3.5 and RC_FRET (dCTP)_ < 3.7 for Lu-Rong and RC_FRET (dUTP)_ > 3.3 and RC_FRET (dGTP)_ < 3.8 for Chuan-Bei-Mu. *: Unsuccessful amplification and bi-directional sequencing in three samples of the Lu-Rong decoction pieces due to the low PCR yield.

**Table 2:** Results of the relative change in the FRET ratio in patented Chinese drugs.

Patented Chinese drugs	RC_FRET (dUTP)_	RC_FRET (dGTP)_	dispensatory label	CCP-based FRET
She Dan Chuan Bei Ruan Jiao Nang	12.54	2.74	Chuan-Bei-Mu	Authentic
Er Mu Ning Sou Wan	6.70	1.44	Chuan-Bei-Mu	Authentic
Bai He Gu Jing Wan^a^	3.01	5.62	Chuan-Bei-Mu	Adulterant
Yang Yin Qing Fei Wan	2.90	4.34	Chuan-Bei-Mu	Adulterant
Bai He Gu Jing Wan^b^	3.16	4.92	Chuan-Bei-Mu	Adulterant
Fu Fang Chuan Bei Jing Pian	3.74	4.07	Chuan-Bei-Mu	Mixture
Yin Er Jian Pi San	14.58	5.35	Chuan-Bei-Mu	Mixture
She Dan Chuan Bei Sang	3.23	3.49	Chuan-Bei-Mu	Unknown
Zhi Sou Hua Tan Wan	3.04	1.98	Chuan-Bei-Mu	Unknown
Qing Yin Wan	3.21	3.24	Chuan-Bei-Mu	Unknown
Ju Hong Wan^c^	2.95	6.51	Zhe-Bei-Mu	Authentic
Ju Hong Wan^b^	2.91	7.23	Zhe-Bei-Mu	Authentic
Jin Sang San Jie Wan	3.02	10.05	Zhe-Bei-Mu	Authentic
Tie Die Wan	3.11	7.20	Zhe-Bei-Mu	Authentic
Wu Bei San	3.26	8.86	Zhe-Bei-Mu	Authentic
Huang Shi Xiang Sheng Wan	2.17	3.07	Zhe-Bei-Mu	Unknown

^a^Concentrated pill.

^b^Water-honey pill; and

^c^Honey pill.
