# Supplementary material for: Fluorescence Visual Detection of Herbal Product Substitutions at Terminal Herbal Markets by CCP-based FRET technique
Source: Sci Rep. 2016 Oct 21;6:35540. doi: 10.1038/srep35540 (PMC5073245; doi:10.1038/srep35540)
Supplement: Supplementary Information [file srep35540-s1.doc]

**Supplementary Information**

**Fluorescence Visual Detection of Herbal Product Substitutions at Terminal Herbal Markets by CCP-based FRET technique**

Chao Jiang^1,2^, Yuan Yuan^1^*, Guang Yang^1^, Yan Jin^1^, Libing Liu^3^, Yuyang Zhao^1^ & Luqi Huang^1^*

1 State Key Laboratory Breeding Base of Dao-di Herbs, National resource center for Chinese Materia Medica, China Academy of Chinese Medical Sciences, Beijing, 100700, P.R. China

2 Beijing Area Major Laboratory of Protection and Utilization of Traditional Chinese Medicine, College of resources, Beijing Normal University, Beijing, 100875, P.R. China

3 Beijing National Laboratory for Molecular Sciences, Key Laboratory of Organic Solids, Institute of Chemistry, Chinese Academy of Sciences, Beijing 100190, P. R. China

**Address for Correspondence**

Prof. Dr. Luqi Huang & Dr Yuan Yuan

State Key Laboratory Breeding Base of Dao-di Herbs, National resource center for Chinese Materia Medica, China Academy of Chinese Medical Sciences, Beijing, 100700, P.R. China

E-mail: [huangluqi01@126.com](mailto:huangluqi01@126.com) (LQ Huang); [yyuan0732@gmail.com](mailto:yyuan0732@gmail.com) (Y Yuan);

Tel: +86 10 64014411-2851

Fax: +86 10 64013996

**Materials and instruments.** PFP was synthesized according to a procedure described in the literature ^1^. Inorganic (yeast) pyrophosphatase shrimp alkaline phosphatase and exonuclease I were purchased from New England Biolabs, Inc. Taq DNA polymerase was purchased from Takara Biotechnology (Dalian) Co., Ltd. N-2-Hydroxyethyl piperazine-N-2-ethanesulfonic acid (HEPES) and DMSO were purchased from Merck Co., Ltd. Fluorescein-12-dUTP (dUTP-Fl), fluorescein-12-dCTP (dCTP-Fl) and fluorescein-12-dGTP (dGTP-Fl) were purchased from Perkin Elmer. The primers were synthesized by Sangon Biotech Co., Ltd and purified using the ULTRA Polyacrylamide Gel Electrophoresis method. MilliQ water (18.2 MΩ) was obtained from an Ultrapure water system (Millipore Inc.). Double distilled water (ddH_2_O) was autoclaved at 120 °C for 15 min using the MilliQ water.

**Primer design:** The PCR primers used in the experiments are described in Tables S5 and S6. Primers were designed using Primer Premier Version 5.0 software (<http://www.premierbiosoft.com/crm/jsp/com/pbi/crm/clientside/ProductList.jsp>). The SNP site was located at the 3’-terminus of the single-base extension (SBE) primer. The Tm of the primer was 50-60 °C, and self-complementarity was avoided.

**Patented Chinese drug screening**: Sixteen patented Chinese drugs containing fritillaria ingredients, She Dan Chuan Bei Ruan Jiao Nang, Qing Yin Wan, Ju Hong Wan (water-honey pill), Er Mu Ning Sou San, Jin Sang San Jie Wan, Tie Di Wan, Huang Shi Xiang Sheng Wan, Wu Bei San, She Dan Chuan Bei San, Fu Fang Chuan Bei Jing Pian, Bai He Gu Jin Wan (concentrated pill), Yang Yin Qing Fei Wan, Yin Er Jian Pi San, Bai He Gu Jin Wan (water-honey pill), Zhi Sou Hua Tan Wan, and Ju Hong Wan (honey pill), were used (details in Table S4). Total DNA was extracted according to the procedure in the literature. PCR amplification and the SBE reaction were conducted using the fritillaria authentication primers and the corresponding fluorescein-12 labeled dNTPs. Upon the addition of PFP to the single-base primer extension products of these DNA samples, the resonance energy transfer with an excitation wavelength of 380 nm was measured to determine the botanical origin. The FRET ratios (I_530 nm_/I_425 nm_) were calculated after the PFP addition.

**Results**

**Sensitivity of the visual FRET-based assay:** The FRET ratios (I_530 nm_/I_425 nm_) for an excitation wavelength of 380 nm from solutions containing CCP and extension products of the fritillaria amplification products obtained by PCR amplification of mixtures with various percentages of authentic (*Fritillaria cirrhosa*) and adulterant CBM (*F. ussuriensis*) genomic DNA using 1F/1R primers were measured after the addition of PFP. Mixtures containing a series of *F. ussuriensis* percentages, ranging from 0, 2, 5, 10, 20, 30, 40, 50, 90 to 100%, were used. The relationship between the FRET ratio and the regression curve is shown in Figure S1.

**Sequencing result of Lu-Rong decoction piece samples:** All Lu-Rong decoction piece samples were amplified with the primer pair 2F/2R, and the PCR products were sequenced using the ABI 3730 xl DNA sequencers. Consensus sequences were searched against the NCBI nucleotide database using the BLASTn program (Table S7). The sequencing chromatogram of suspected mixture samples is shown in Figure S2.

**Pharmacognosy authentication result of Chuan-Bei-Mu decoction piece samples:** All Chuan-Bei-Mu decoction piece samples were morphologically authenticated by 3 trained pharmacognostic experts, and the consensus conclusion from at least two experts was deemed the morphological identification result (Table S8).

Samples: Fresh plants, medicinal materials and patented Chinese drugs were collected (Tables S1, S2, and S3).

Supplementary Table 1. Chinese medicinal material samples used in this study

| **Code** | **Trade name** | **Collected area** | ***Species*** |
| --- | --- | --- | --- |
| Fritillaria | | | |
| BM-1 | Song Bei | Hefei, Anhui prov. | *Fritillaria cirrhosa* |
| BM-2 | Song Bei | Hefei, Anhui prov. | *F. unibracteata* |
| BM-3 | Qing Bei | Hefei, Anhui prov. | *F. delavayi* |
| BM-4 | Lu Bei | Hefei, Anhui prov. | *F. przewalskii* |
| BM-5 | Taibai Beimu | Hefei, Anhui prov. | *F. taipaiensis* |
| BM-6 | Wabu Beimu | Hefei, Anhui prov. | *F. wabuensis* |
| BM-7 | Xinjiang Beimu | Hefei, Anhui prov. | *F. walujewii* |
| BM-8 | Yibeimu | Hefei, Anhui prov. | *F. pallidiflora* |
| BM-9 | Ping Bei (big) | Hefei, Anhui prov. | *F. ussuriensis* |
| BM-10 | Ping Bei (small) | Hefei, Anhui prov. | *F. ussuriensis* |
| BM-11 | Xiaomi Bei | Hefei, Anhui prov. | *F. ussuriensis* |
| BM-12 | Xiang Bei | Hefei, Anhui prov. | *F. thunbergii* |
| BM-13 | Yuanbao Bei | Hefei, Anhui prov. | *F. thunbergii* |
| BM-14 | Xiaodong Bei | Hefei, Anhui prov. | *F. thunbergii var. Chekiangensis* |
| BM-15 | Wan Beimu | Hefei, Anhui prov. | *F. anhuiensis* |
| BM-16 | Yilun Beimu | Hefei, Anhui prov. | *F. maximowiczii* |
| BM-17 | Hubei Beimu | Hefei, Anhui prov. | *F. hupehensis* |
| Cervus | | | |
| LR-1 | sika deer | Hefei, Anhui prov. | *Cervus nippon* |
| LR-2 | red deer | Hefei, Anhui prov. | *C. elaphus* |
| LR-3 | sambar | Beijing | *C. unicolor* |
| LR-4 | reindeer | Beijing | *Rangifer tarandus* |
| LR-5 | white-lipped deer | Hefei, Anhui prov. | *C. albirostris* |
| LR-6 | Pere David’s deer | Beijing | *Elaphurus davidianus* |
| LR-7 | fallow deer | Beijing | *Dama dama* |
| LR-8 | mute deer | Hefei, Anhui prov. | *Odocoileus hemionus* |
| LR-9 | roe deer | Beijing | *Capreolus capreolus* |

Supplementary Table 2 Chuan-Bei-Mu decoction piece samples used in this study

| **Code** | **Origin area** | **Collected from** | **Collected date** | **Prices (￥/g)** |
| --- | --- | --- | --- | --- |
| CB0001 | Handan, Hebei prov. | Hospital | 2013.09 | 5.63 |
| CB0002 | Handan, Hebei prov. | Hospital | 2013.09 | 1.5 |
| CB0003 | Handan, Heibei prov. | Hospital | 2013.09 | 4.8 |
| CB0004 | Taibei, Taiwan prov. | pharmacy | 2013.09 | 0.5 |
| CB0005 | Handan, Hebei prov. | pharmacy | 2013.09 | 0.3 |
| CB0006 | Handan, Hebei prov. | pharmacy | 2013.09 | 0.4 |
| CB0007 | Taibei, Taiwan prov. | pharmacy | 2013.09 | 0.83 |
| CB0008 | Hualian, Taiwan prov. | pharmacy | 2013.09 | 0.25 |
| CB0009 | Handan, Hebei prov. | pharmacy | 2013.09 | 5.2 |
| CB0010 | Xinbei, HongKong | pharmacy | 2013.09 | 2.77 |
| CB0011 | Guiyang, Guizhou prov. | pharmacy | 2013.09 | 1.5 |
| CB0012 | Guiyang, Guizhou prov. | pharmacy | 2013.09 | 3.7 |
| CB0013 | Guiyang, Guizhou prov. | pharmacy | 2013.09 | 3.8 |
| CB0014 | Guiyang, Guizhou prov. | pharmacy | 2013.09 | 0.5 |
| CB0015 | Chengdu, Sicuan prov. | pharmacy | 2013.09 | 4.8 |
| CB0016 | Beijing | pharmacy | 2013.09 | 5 |
| CB0017 | Chengdu, Sicuan prov. | Hospital | 2013.09 | 3.86 |
| CB0018 | Chengdu, Sicuan prov. | Hospital | 2013.09 | 5 |
| CB0019 | Chengdu, Sicuan prov. | pharmacy | 2013.09 | 5.5 |
| CB0020 | Chengdu, Sicuan prov. | pharmacy | 2013.09 | 4.2 |
| CB0021 | Chengdu, Sicuan prov. | pharmacy | 2013.09 | 4.08 |
| CB0022 | Tianjin | Hospital | 2013.09 | 0.46 |
| CB0023 | Tianjin | pharmacy | 2013.09 | 1.2 |
| CB0024 | Tianjin | pharmacy | 2013.09 | 4.8 |
| CB0025 | Tianjin | Hospital | 2013.09 | 2.84 |
| CB0026 | Tianjin | Hospital | 2013.09 | 0.22 |
| CB0027 | Tianjin | hospital | 2013.09 | 0.6 |
| CB0028 | Guangtao, Hebei prov. | pharmacy | 2013.09 | 0.35 |
| CB0029 | Guangtao, Hebei prov. | pharmacy | 2013.1 | 0.3 |
| CB0030 | Guangtao, Hebei prov. | hospital | 2013.1 | 0.16 |
| CB0031 | Xinjie,HongKong | company | 2013.1 | 2.9 |
| CB0032 | Yuanlan, HongKong | drug market | 2013.1 | 2.28 |
| CB0033 | Yuanlan, HongKong | company | 2013.1 | 1.9 |
| CB0034 | Baotou, Inner Mongolia prov. | hospital | 2013.1 | 3.21 |
| CB0035 | Baotou, Inner Mongolia prov. | hospital | 2013.1 | 1.8 |
| CB0036 | Zhuhai, Guangdong prov. | pharmacy | 2013.1 | 0.4 |
| CB0037 | Baotou, Inner Mongolia prov. | hospital | 2013.09 | 7.48 |
| CB0038 | Macau | company | 2013.1 | 0.57 |
| CB0039 | Baotou, Inner Mongolia prov. | pharmacy | 2013.1 | 2 |
| CB0040 | Macau | company | 2013.1 | 0.6 |
| CB0041 | Zhuhai, Guangdong prov. | pharmacy | 2013.1 | 5 |
| CB0042 | Zhuhai, Guangdong prov. | pharmacy | 2013.1 | 4.3 |
| CB0043 | Macau | pharmacy | 2013.1 | 0.76 |
| CB0044 | Nanning, Guangxi prov. | pharmacy | 2013.09 | 6 |
| CB0045 | Nanning, Guangxi prov. | hospital | 2013.1 | 0.81 |
| CB0046 | Nanning, Guangxi prov. | hospital | 2013.1 | 5.06 |
| CB0047 | Nanning, Guangxi prov. | pharmacy | 2013.09 | 8.2 |
| CB0048 | Nanning, Guangxi prov. | pharmacy | 2013.1 | 6 |
| CB0049 | Wuhan,Hubei prov. | hospital | 2013.1 | 6.24 |
| CB0050 | Baotou, Inner Mongolia prov. | pharmacy | 2013.1 | 3.5 |
| CB0051 | Nanning, Guangxi prov. | hospital | 2013.09 | 5.16 |
| CB0052 | Baotou, Inner Mongolia prov. | pharmacy | 2013.1 | 3 |
| CB0053 | Wuhan,Hubei prov. | hospital | 2013.09 | 5.4 |
| CB0054 | Lanzhou, Gansu prov. | hospital | 2013.1 | 3.28 |
| CB0055 | Lanzhou, Gansu prov. | hospital | 2013.11 | 2.88 |
| CB0056 | Lanzhou, Gansu prov. | pharmacy | 2013.11 | 4.8 |
| CB0057 | Lanzhou, Gansu prov. | hospital | 2013.11 | 0.4 |
| CB0058 | Lanzhou, Gansu prov. | pharmacy | 2013.11 | 0.5 |
| CB0059 | Lanzhou, Gansu prov. | pharmacy | 2013.11 | 2 |

Supplementary Table 3 Lu-Rong decoction piece samples used in this study

| **Code** | **Origin area** | **Collected from** | **Collected date** | **Specifications*** |
| --- | --- | --- | --- | --- |
| LR-001 | Bozhou, Anhui prov. | pharmacy | 2014.07.28 | BoS |
| LR-002 | Bozhou, Anhui prov. | pharmacy | 2014.07.28 | BoS |
| LR-003 | Bozhou, Anhui prov. | pharmacy | 2014.10.03 | BlS |
| LR-004 | Bozhou, Anhui prov. | pharmacy | 2014.10.03 | PS |
| LR-005 | Bozhou, Anhui prov. | pharmacy | 2014.10.03 | BoS |
| LR-006 | Bozhou, Anhui prov. | pharmacy | 2014.10.03 | WS |
| LR-007 | Bozhou, Anhui prov. | pharmacy | 2014.10.03 | WS |
| LR-008 | Beijing | pharmacy |  | PS |
| LR-009 | Guangzhou, Guangdong prov. | pharmacy | 2014.10.06 | WS |
| LR-010 | Guangzhou, Guangdong prov. | pharmacy | 2014.10.06 | PS |
| LR-011 | Kunming, Yunan prov. | pharmacy | 2014.04.18 | PS |
| LR-012 | Guangzhou, Guangdong prov. | pharmacy | 2014.10.06 | BlS |
| LR-013 | Changsha, Hunan prov. | pharmacy | 2014.04.10 | PS |
| LR-014 | Guangzhou, Guangdong prov. | pharmacy | 2014.10.06 | PS |
| LR-015 | Guangzhou, Guangdong prov. | pharmacy | 2014.10.06 | BoS |
| LR-016 | Guangzhou, Guangdong prov. | pharmacy | 2014.10.06 | BlS |
| LR-017 | Bozhou, Anhui prov. | pharmacy | 2014.04.10 | BlS |
| LR-018 | Bozhou, Anhui prov. | pharmacy | 2014.04.18 | BlS |
| LR-019 | Guangzhou, Guangdong prov. | pharmacy | 2014.10.06 | BlS |
| LR-020 | Bozhou, Anhui prov. | pharmacy | 2014.02.21 | PS |
| LR-021 | Yulin, Guangxi prov. | pharmacy | 2014.10.06 | PS |
| LR-022 | Yulin, Guangxi prov. | pharmacy | 2014.10.06 | PS |
| LR-023 | Bozhou, Anhui prov. | pharmacy | 2014.03.10 | PS |
| LR-024 | Bozhou, Anhui prov. | pharmacy | 2014.03.10 | BoS |
| LR-025 | Bozhou, Anhui prov. | pharmacy | 2014.03.10 | BoS |
| LR-026 | Beijing | pharmacy | 2014.08.12 | BlS |
| LR-027 | Beijing | pharmacy | 2014.08.12 | BlS |
| LR-028 | Beijing | pharmacy | 2014.08.12 | WS |
| LR-029 | Beijing | pharmacy | 2014.08.12 | WS |
| LR-030 | Beijing | pharmacy | 2014.08.12 | WS |
| LR-031 | Bozhou, Anhui prov. | pharmacy | 2014.03.11 | WB |
| LR-032 | Bozhou, Anhui prov. | pharmacy | 2014.03.11 | WB |
| LR-033 | Bozhou, Anhui prov. | pharmacy | 2014.03.11 | WB |
| LR-034 | Bozhou, Anhui prov. | pharmacy | 2014.07.28 | WB |
| LR-035 | Bozhou, Anhui prov. | pharmacy | 2014.08.01 | WB |
| LR-036 | Urumqi, Xinjiang prov. | pharmacy | 2014.06.28 | WB |
| LR-037 | Chongqin | pharmacy | 2014.03.05 | WB |
| LR-038 | Urumqi, Xinjiang prov. | pharmacy | 2014.06.28 | WB |
| LR-039 | Urumqi, Xinjiang prov. | pharmacy | 2014.06.28 | WB |
| LR-040 | Urumqi, Xinjiang prov. | pharmacy | 2014.06.28 | WB |
| LR-041 | Bozhou, Anhui prov. | pharmacy | 2014.08.01 | WB |
| LR-042 | Bozhou, Anhui prov. | pharmacy | 2014.07.28 | WB |
| LR-043 | Bozhou, Anhui prov. | pharmacy | 2014.08.01 | WB |
| LR-044 | Urumqi, Xinjiang prov. | pharmacy | 2014.06.28 | WB |
| LR-045 | Shenyang, Liaoning prov. | pharmacy | 2014.08.01 | PS |
| LR-046 | Beijing | pharmacy | 2014.02.21 | PS |
| LR-047 | Beijing | pharmacy | 2014.02.21 | PS |
| LR-048 | Beijing | pharmacy | 2014.02.21 | PS |
| LR-049 | Beijing | pharmacy | 2014.02.21 | PS |
| LR-050 | Beijing | pharmacy | 2014.02.21 | PS |
| LR-051 | Beijing | pharmacy | 2014.02.22 | PS |
| LR-052 | Beijing | pharmacy | 2014.02.22 | WS |
| LR-053 | Beijing | pharmacy | 2014.02.22 | WS |

* WB: Whole branch, WS: wax-liked slice, PS: powder slice, BlS: blood slice, BoS: bone slice.

Supplementary Table 4 Chinese patent drug samples used in this study

| Code | Patented Chinese drugs | Identification method in Chinese Pharmacopoeia | Raw materials number | Form | Lot number |
| --- | --- | --- | --- | --- | --- |
| ZCY01 | She Dan Cuan Bei Ruan Jiao Nang | TLC | 2 | soft capsule | 20130009 |
| ZCY02 | She Dan Cuan Bei San | TLC, microscopy | 2 | podwer | 13032 |
| ZCY03 | Fu Fang Cuan Bei Jing Pian | microscopy | 8 | Tablet | 13121078 |
| ZCY04 | Bai He Gu Jing Wan | - | 10 | concentrated pill | 52141008 |
| ZCY05 | Yang Yin Qing Fei Wan | - | 8 | honeyed pill | 3035291 |
| ZCY06 | Yin Er Jian Pi San | Not List | 7 | podwer | 120803 |
| ZCY07 | Bai He Gu Jing Wan | microscopy | 8 | water-honeyed pill | 131001116 |
| ZCY08 | Zhi Sou Hua Tang Wan | - | 25 | water-bindered pill | 20130221 |
| ZCY09 | Ju Hong Wan | - | 8 | honeyed pill | 130901 |
| ZCY10 | Qing Yin Wan | TLC, microscopy | 8 | honeyed pill | 3013083 |
| ZCY11 | Ju Hong Wan | - | 8 | water-honeyed pill | 131119 |
| ZCY12 | Er Mu Ning Sou Wan | microscopy | 12 | honeyed pill | 4015077 |
| ZCY13 | Jin Sang San Jie Wan | Not List | 16 | water-bindered pill | 201210192 |
| ZCY14 | Tie Di Wan | microscopy | 10 | honeyed pill | 13030022 |
| ZCY15 | Huang Si Xiang Sheng Wan | HPLC | 12 | water-bindered pill | 131110 |
| ZCY16 | Wu Bei San | microscopy | 3 | podwer | 110901 |

Table S5 Amplification primers used in this study

| **application** | **Primer Name** | **Primer sequences (5’→3’)** | **Amplicons Size(bp)** | **Annealing Tm(°C)** |
| --- | --- | --- | --- | --- |
| fritillaries | CB-F | GCGAAATGCGATACTTGGTGTGA | 275 | 56 |
|  | CB-F | GTGTCCCCGCCTGACCTGG |
| deer antler | LR-F | AATATTACTAGTATTATTCGCACCAGA | 424 | 56 |
|  | LR-R | TTCAGAATAGGCATTGGCTG |

| **application** | **Primer Name** | **Primer sequences (5’→3’)** | **Annealing Tm(°C)** | **Incorporated**  **dye-dNTP** | |
| --- | --- | --- | --- | --- | --- |
|  |  |  |  | **Authentic** | **Adulterant** |
| fritillaries | BM-FRET | TCTCCGCATCCGTGACCGC | 56 | Fl-dUTP | Fl-dGTP |
| deer antler | LR-FRET | CCCAACAAACTAGGAGGAGTC | 60 | Fl-dCTP | Fl-dUTP |

Table S6 Single-base extension primers used in this study

Supplementary Table 7. Sequencing results of Lu-Rong decoction piece samples

| Samples | CCP-FRET assay | BLAST | Samples | CCP-FRET assay | BLAST |
| --- | --- | --- | --- | --- | --- |
| LR-3 | Mixture | Cervus elaphus | LR-4 | Authentic | Cervus elaphus |
| LR-11 | Mixture | Rangifer tarandus | LR-5 | Authentic | Cervus elaphus |
| LR-18 | Mixture | Rangifer tarandus | LR-12 | Authentic | Cervus elaphus |
| LR-23 | Mixture | Rangifer tarandus | LR-15 | Authentic | Cervus elaphus |
| LR-1 | Adulterant | Rangifer tarandus | LR-17 | Authentic | Cervus elaphus |
| LR-2 | Adulterant | Odocoileus hemionus | LR-19 | Authentic | Cervus elaphus |
| LR-6 | Adulterant | Przewalskium albirostris | LR-22 | Authentic | Cervus elaphus |
| LR-7 | Adulterant | Rangifer tarandus | LR-31 | Authentic | Cervus nippon |
| LR-8 | Adulterant | Rangifer tarandus | LR-32 | Authentic | Cervus nippon |
| LR-9 | Adulterant | Rangifer tarandus | LR-33 | Authentic | Cervus nippon |
| LR-10 | Adulterant | Rangifer tarandus | LR-34 | Authentic | Cervus elaphus |
| LR-13 | Adulterant | Rangifer tarandus | LR-35 | Authentic | Cervus nippon |
| LR-14 | Adulterant | Rangifer tarandus | LR-36 | Authentic | Cervus elaphus |
| LR-16 | Adulterant | Rangifer tarandus | LR-37 | Authentic | Cervus nippon |
| LR-20 | Adulterant | Rangifer tarandus | LR-38 | Authentic | Cervus elaphus |
| LR-21 | Adulterant | Rangifer tarandus | LR-39 | Authentic | Cervus nippon |
| LR-24 | Adulterant | Unsuccess | LR-40 | Authentic | Cervus nippon |
| LR-25 | Adulterant | Unsuccess | LR-41 | Authentic | Cervus nippon |
| LR-26 | Adulterant | Unsuccess | LR-42 | Authentic | Cervus nippon |
| LR-27 | Adulterant | Capreolus pygargus | LR-43 | Authentic | Cervus elaphus |
| LR-28 | Adulterant | Capreolus pygargus | LR-44 | Authentic | Cervus elaphus |
| LR-29 | Adulterant | Capreolus pygargus | LR-45 | Authentic | Cervus elaphus |
| LR-30 | Adulterant | Capreolus pygargus | LR-49 | Authentic | Cervus nippon |
| LR-46 | Adulterant | Rucervus eldi | LR-50 | Authentic | Cervus elaphus |
| LR-47 | Adulterant | Rusa unicolor | LR-53 | Authentic | Cervus elaphus |
| LR-48 | Adulterant | Elaphurus davidianus | | |  |
| LR-51 | Adulterant | Alces alces | |  |  |
| LR-52 | Adulterant | Rangifer tarandus | |  |  |

Supplementary Table 8. Morphological identification results of Chuan-Bei-Mu decoction piece samples

| Samples | Morphological identification* | CCP-FRET assay | Samples | Morphological identification* | CCP-FRET assay |
| --- | --- | --- | --- | --- | --- |
| CB0001 | SB&QB ( Authentic) | Authentic | CB0002 | PB (Adulterant) | Adulterant |
| CB0009 | SB ( Authentic) | Authentic | CB0004 | PB (Adulterant) | Adulterant |
| CB0010 | LB ( Authentic) | Authentic | CB0005 | PB (Adulterant) | Adulterant |
| CB0013 | QB ( Authentic) | Authentic | CB0007 | PB (Adulterant) | Adulterant |
| CB0015 | SB&QB ( Authentic) | Authentic | CB0008 | ZB (Adulterant) | Adulterant |
| CB0016 | SB ( Authentic) | Authentic | CB0014 | PB (Adulterant) | Adulterant |
| CB0017 | SB ( Authentic) | Authentic | CB0022 | PB (Adulterant) | Adulterant |
| CB0018 | SB ( Authentic) | Authentic | CB0023 | Unknown | Adulterant |
| CB0019 | SB ( Authentic) | Authentic | CB0026 | PB (Adulterant) | Adulterant |
| CB0020 | SB ( Authentic) | Authentic | CB0027 | PB (Adulterant) | Adulterant |
| CB0021 | SB ( Authentic) | Authentic | CB0028 | PB (Adulterant) | Adulterant |
| CB0041 | SB&QB ( Authentic) | Authentic | CB0029 | PB (Adulterant) | Adulterant |
| CB0044 | SB ( Authentic) | Authentic | CB0030 | PB (Adulterant) | Adulterant |
| CB0046 | SB&QB ( Authentic) | Authentic | CB0032 | PB (Adulterant) | Adulterant |
| CB0048 | SB ( Authentic) | Authentic | CB0033 | PB (Adulterant) | Adulterant |
| CB0049 | SB ( Authentic) | Authentic | CB0034 | YB&XB (Adulterant) | Adulterant |
| CB0051 | SB ( Authentic) | Authentic | CB0035 | PB&XB (Adulterant) | Adulterant |
| CB0053 | SB ( Authentic) | Authentic | CB0036 | PB (Adulterant) | Adulterant |
| CB0054 | QB ( Authentic) | Authentic | CB0037 | XB (Adulterant) | Adulterant |
| CB0056 | QB ( Authentic) | Authentic | CB0038 | PB (Adulterant) | Adulterant |
| CB0003 | PB (Adulterant) | Mixture | CB0039 | PB (Adulterant) | Adulterant |
| CB0024 | LB&PB (Mixture) | Mixture | CB0040 | ZB (Adulterant) | Adulterant |
| CB0025 | LB&ZQB (Mixture) | Mixture | CB0043 | ZB (Adulterant) | Adulterant |
| CB0047 | SB&YB (Mixture) | Mixture | CB0045 | PB (Adulterant) | Adulterant |
| CB0055 | PB (Adulterant) | Mixture | CB0050 | PB (Adulterant) | Adulterant |
|  |  |  | CB0052 | PB (Adulterant) | Adulterant |
|  |  |  | CB0057 | PB (Adulterant) | Adulterant |
|  |  |  | CB0058 | PB (Adulterant) | Adulterant |
|  |  |  | CB0059 | XB (Adulterant) | Adulterant |

*SB: Song Bei; QB: Qing Bei; LB: Lu Bei; PB: Pei Bei; ZB: Zhe Bei; XB: Xinjiang Beimu; YB: Yi Beimu; ZQB: Xizhang Beimu

Figure S1. Relationship between the FRET ratio (I_530 nm_/I_425 nm_) and the adulterant percentage: For the mean, error bars indicate ± s.d. (n=3). All the experiments were performed in HEPES buffer solution (25 mM, pH 7.5). The excitation wavelength was 380 nm. [dCTP-Fl] = 5×10^-8^ M, [dUTP-Fl] = 5×10^-8^ M, and [CCP] = 1.5×10^-6^ M.

**
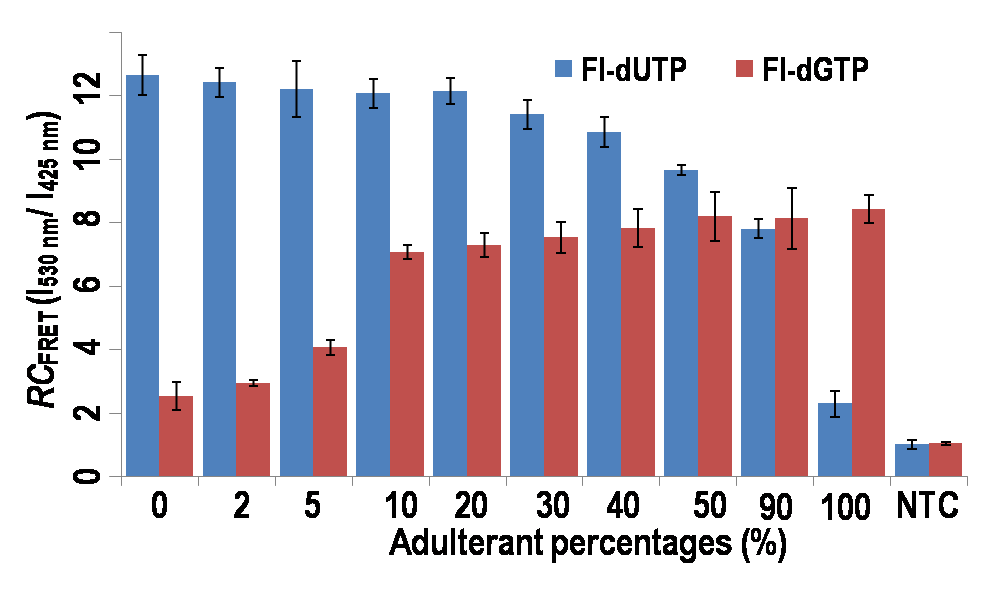
**

Figure S2. DNA sequencing analysis of four suspected mixture sample

**
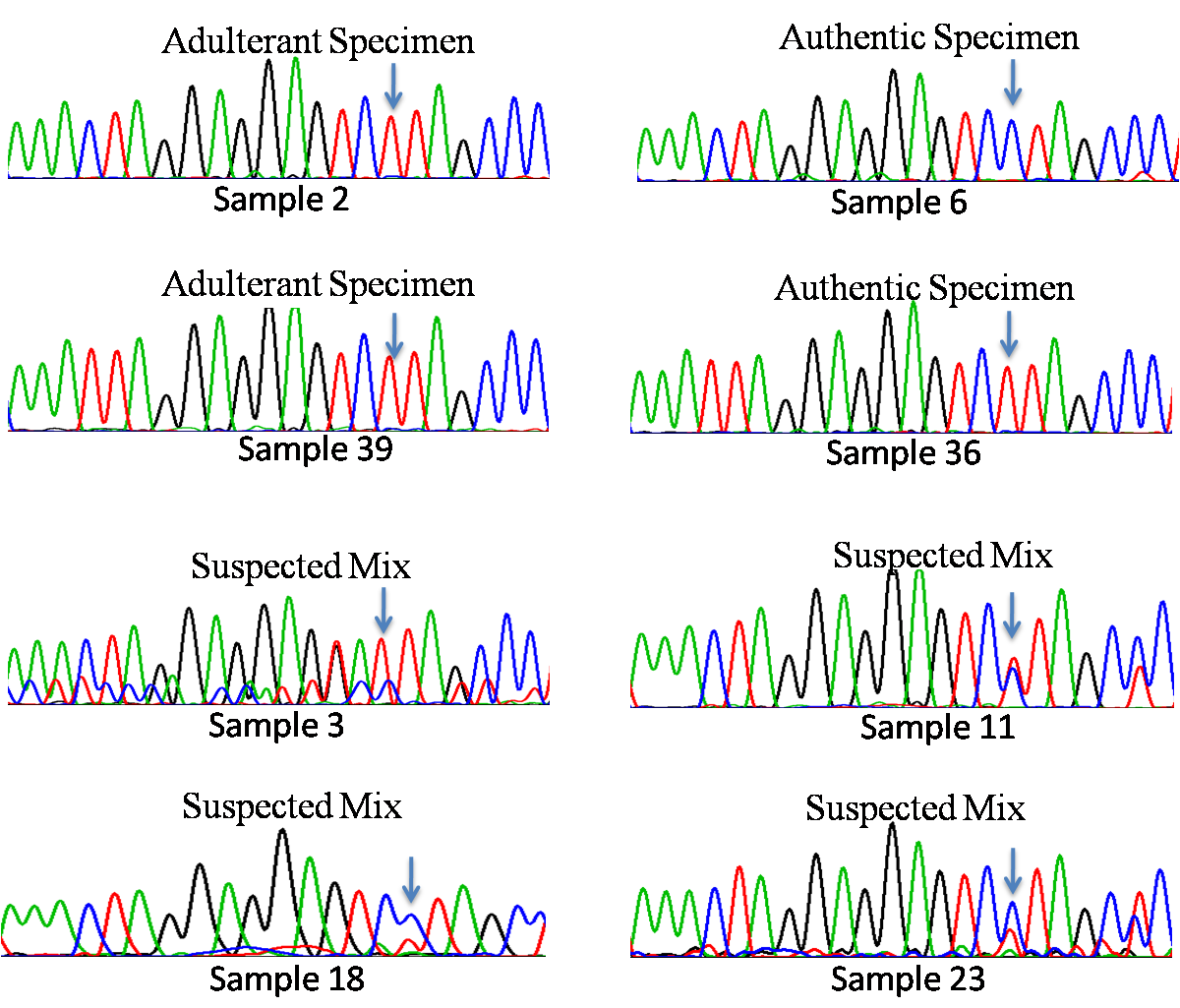
**

Figure S3. (a) Relative fluorescence resonance energy transfer (FRET) ratio of PFP to fluorescein RC_FRET_ (I_530_ nm/I_425_ nm) in Chuan-Bei-Mu specimens using Fl-dUTP (y axis) in an SBE reaction. (b) PCR-RFLP as reference standard method to identify Chuan-Bei-Mu specimens. M: DL 2000 DNA marker; lane 1 to lane 20 were Chuan-Bei-Mu decoction piece samples in table 2. Lane 81 to lane 48 were Chuan-Bei-Mu samples from An’hui Institute for Food and Drug Control. NTC: No temple control.

**
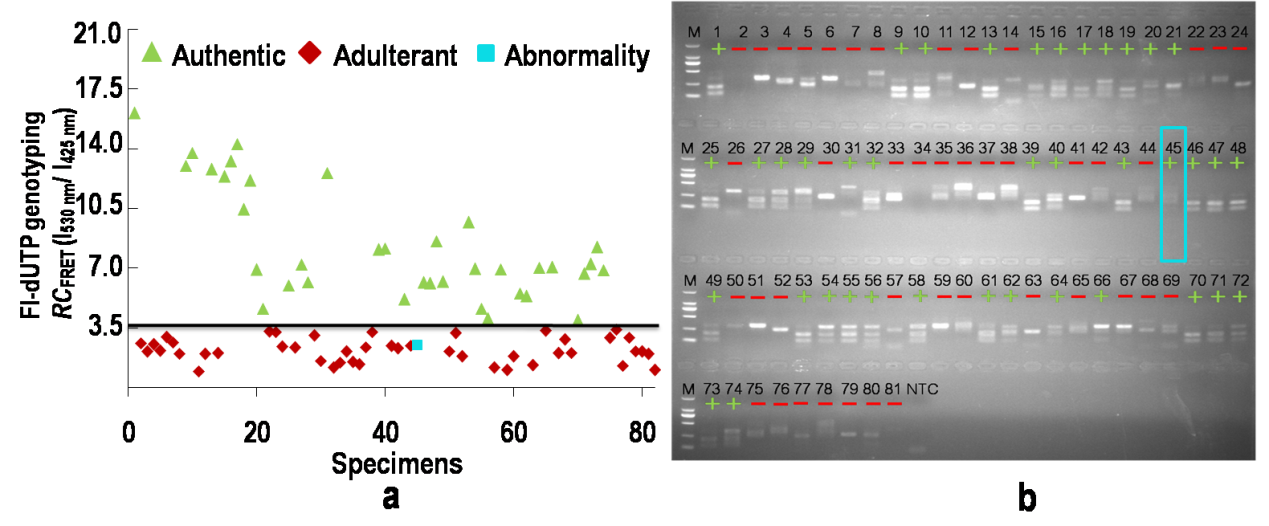
**

**References**

1. Duan, X.R. *et al.* Single-Nucleotide Polymorphism (SNP) Genotyping Using Cationic Conjugated Polymers in Homogeneous Solution. *Nat. Protoc.* **4**, 984-991 (2009).
